# Anti-lysoganglioside and other anti-neuronal autoantibodies in post-treatment Lyme Disease and Erythema Migrans after repeat infection

**DOI:** 10.1016/j.bbih.2019.100015

**Published:** 2020-01-07

**Authors:** Brian A. Fallon, Barbara Strobino, Sean Reim, Julie Stoner, Madeleine W. Cunningham

**Affiliations:** a Columbia Psychiatry, Columbia University Irving Medical Center, New York, USA; b New York State Psychiatric Institute, 1051 Riverside Drive, New York, USA; c Department of Microbiology and Immunology, University of Oklahoma Health Sciences Center, Oklahoma City, USA; d Department of Biostatistics, University of Oklahoma Health Sciences Center, Oklahoma City, USA

**Keywords:** Lyme disease, Antineuronal antibodies, Lysoganglioside, CaMKinase II

## Abstract

**Background:**

Molecular mimicry targeting neural tissue has been reported after *Borrelia burgdorferi*(*Bb*) infection. Herein, we investigate whether antineuronal autoantibodies are increased and whether antibody-mediated signaling of neuronal cells is elevated in a cohort of symptomatic adults with a history of Lyme Disease (LD).

**Methods:**

Participants (n ​= ​179) included 24 with recent Erythema Migrans (EM) without prior LD, 8 with recent EM and prior LD (EM ​+ ​prior LD), 119 with persistent post-treatment LD symptoms (PTLS), and 28 seronegative endemic controls with no prior LD history. Antineuronal immunoglobulin G (IgG) titers were measured by standard ELISA and compared with mean titers of normal age-matched sera against lysoganglioside, tubulin, and dopamine receptors (D1R and D2R). Antibody-mediated signaling of calcium calmodulin dependent protein kinase II (CaMKII) activity in a human neuronal cell line (SK-N-SH) was identified in serum.

**Results:**

EM ​+ ​prior LD cases had higher antibody titers than controls for anti-lysoganglioside GM1 (p ​= ​0.002), anti-tubulin (p ​= ​0.03), and anti-D1R (p ​= ​0.02), as well as higher expression in the functional antibody-mediated CaMKII Assay (p ​= ​0.03). The EM cases with no prior history showed no significant differences on any measures. The PTLS cases demonstrated significantly higher titers (p ​= ​0.01) than controls on anti-lysoganglioside GM1, but not for the other measures.

**Conclusion:**

The finding of elevated anti-neuronal autoantibodies in our small sample of those with a prior history of Lyme disease but not in those without prior Lyme disease, if replicated in a larger sample, suggests an immune priming effect of repeated infection; the CaMKII activation suggests that antineuronal antibodies have functional significance. The elevation of anti-lysoganglioside antibodies among those with PTLS is of particular interest given the established role of anti-ganglioside antibodies in peripheral and central neurologic diseases. Future prospective studies can determine whether these autoantibodies emerge after *Bb* infection and whether their emergence coincides with persistent neurologic or neuropsychiatric symptoms.

## Highlights

• The EM ​+ ​prior LD group had significantly elevated anti-neuronal antibodies.
• The EM ​+ ​prior LD group had significantly elevated CaM Kinase activation.
• Anti-Lysoganglioside Antibodies are significantly elevated in the PTLS group.
• Prior infection may lead to immune priming and increased autoantibodies.

## 1 Introduction

Lyme disease (*LD*), caused by the spirochete *Borrelia burgdorferi (Bb*), is characterized by dermatologic, rheumatologic, neurologic, and cardiac manifestations. Clinical disease is predominantly caused by the immune response to the spirochete, as *Bb* itself does not produce toxins or extracellular matrix-degrading proteases. Human infection, when treated with antibiotics, most often leads to symptom resolution. However, in about 10–20 percent of treated patients, symptoms persist, most commonly causing fatigue, pain, and/or cognitive complaints; these are commonly referred to as Post-Treatment Lyme Symptoms (PTLS; Steere et al., 2016) or as Post-Treatment Lyme Disease Syndrome (PTLDS; Aucott et al., 2013) if functional impairment is also present. Possible causes of symptom persistence after antibiotic therapy include persistent infection or post-infectious processes such as ongoing immune activation or dysregulated neural networks.

Research in humans with *Bb* infection has demonstrated that the serum contains a variety of autoantibodies that cross-react with human tissue. Some are specific to epitopes on Bb and others are not. In a study of Lyme neuroborreliosis, 29% had anti-*Bb* antibodies that cross-reacted with CNS ganglioside GM1 (García-Moncó et al., 1993). Anti-ganglioside antibodies have been associated with autoimmune-mediated peripheral neuropathies, such as Guillain-Barre syndrome (Yuki, 2012), as well as diverse connective tissue diseases, such as systemic lupus erythematosus and Sjogren’s syndrome (Weiner et al., 2000). Other studies reported that serum from patients with neurologic LD contained anti-*Bb* IgM antibodies that were autoreactive to human peripheral nerve axons (Sigal, 1993). Further, anti-neuronal antibody reactivity has been shown to be much greater among patients with PTLDS than among recovered patients; the antigenic triggers for these autoantibodies were not specified (Chandra et al., 2010). In addition, autoantibodies have been reported against endothelial cell growth factor (ECGF) significantly more often among patients with PTLDS than among recovered patients; anti-ECGF antibodies correlated directly with IL-23 levels (Strle et al., 2014). Other studies have identified altered immunologic profiles in Lyme disease that are associated with chronic symptoms, such as elevated levels of IL23 (Strle et al., 2014) and CCL19 (Aucott et al., 2016) at the time of initial infection. Among PTLDS patients, compared to controls, significantly elevated expression of interferon-α and greater antibody reactivity to brain proteins have been shown; neither biomarker changed significantly after ceftriaxone treatment. Thus, a non-infectious immune-mediated process may be inducing disease (Jacek et al., 2013). A PET brain imaging study of PTLDS demonstrated elevated microglial activation compared to controls (Coughlin et al., 2018), further supporting an inflammatory process in PTLDS.

In vitro and animal studies suggest that anti-*Bb* antibodies may lead to neurologic disease by cross-reactivity. IgG antibodies against *Bb* flagellin cross-reacted with human nerve axons and with HSP-60 of neuroblastoma cells (Fikrig et al., 1993; Yu et al., 1997), and IgG antibodies against *Bb* OspA peptide cross-reacted with human brain neurons, spinal cord, and dorsal root ganglia (Alaedini and Latov, 2005). Further, a mouse study demonstrated that *Bb* infection led to elevated IgM cross reactivity and higher degrees of joint swelling in a strain of mice known to develop autoimmune reactions (Raveche et al., 2005). Raveche et al. further demonstrated sequence homology between *Bb* and group A beta hemolytic streptococcus (GABHS), as well as cross-reactivity of *Bb* antibodies with GABHS M protein and with myosin. Others have shown that rats immunized with *Bb* produced IgM antibodies that cross-reacted with ganglioside-monosialic acid (GM1) and asialo-GM1(Garcia-Monco et al., 1995).

Herein, we present evidence that autoantibodies produced in patients with early Lyme disease and PTLDS cross-react with neuronal antigens potentially leading to Lyme disease sequelae. We selected a group of anti-neuronal autoantibodies that are found in sequelae of GABHS infection for this investigation because of the known sequence homology between *Bb* and GABHS and because of the association of these anti-neuronal autoantibodies with neurologic and neuropsychiatric symptoms. As in rheumatic fever and Sydenham’s chorea, serum from children with acute onset neuropsychiatric disorders after GABHS have been shown in some studies to have elevated anti-neuronal IgG antibodies against lysoganglioside GM1(Kirvan et al., 2006), tubulin (Kirvan et al., 2007), and dopamine receptors D1 and D2 (Ben-Pazi et al., 2013; Cunningham and Cox, 2016). These antibodies develop because of shared antigenic epitopes between the host and GABHS. Further, these antibodies appear to have functional importance as serum from these patients induced neuronal cell signaling through CaMKII activation in a human neuronal cell line (Kirvan et al.,2003, 2006).

We report here the results of the first study examining the hypothesis that the serum of patients with Lyme disease have a greater frequency of specific anti-neuronal autoantibodies and functional neuronal activation compared to community controls without a history of Lyme disease.

## 2 Materials and methods

### 2.1 Population

Stored serum samples from a community serosurvey conducted in hyperendemic LD areas of New York, New Jersey, and Connecticut were examined; the NYS Psychiatric Institute IRB approved the study sample collection and all participants signed informed consent (Strobino et al., 2018). Clinical details of LD history were obtained through questionnaires at the time of the survey and through follow-up telephone contact to obtain additional information. The mean age of the entire study population was 55.8 years (s.d. ​= ​12.4) with slightly more women (58.6%) than men. There were no significant differences between the study case cohorts and controls with respect to age or gender. A total of 179 individual samples were included in this study, drawn from four cohorts. Further information about this community sample has been published (Strobino et al., 2018).

### 2.2 Four cohorts (total n ​= ​179) (see Table 1)

**Table 1:** Description of 4 study cohorts from Lyme endemic areas.

Study cohort	N	Symptoms/Signs at time of serum collection^a^	History of Prior Episodes of LD^b^	Gender (% male)	Mean age (s.d.)
EM cases, No prior LD	24	Clinician diagnosed LD (EM rash) within prior 3 months	Never diagnosed or treated for LD	54.2%	55.9 (13.6)
EM cases, with prior LD	8	Clinician diagnosed LD (EM rash) within prior 3 months	Prior diagnosis and treatment of LD	37.5%	57.4 (11.3)
PTLS^c^	119	Persistent distressing symptoms of fatigue, pain, or cognitive problems attributed to prior LD	Prior diagnosis and treatment of LD with only partial resolution of symptoms	41.2%	55.6 (12.7)
Community controls	28	Healthy or mild non-specific symptoms	Never diagnosed or treated for LD; all 6 tests for *Bb* antibodies are negative.	46.4%	56.0 (10.6)

a Erythema Migrans (EM) is an early sign of infection with Bb; it is diagnosed based on a typical rash (≥2 inches in diameter) acquired in a Lyme endemic region.

b Prior Lyme disease refers to any prior diagnosis and treatment for Lyme disease (not including the current episode) and which may have presented with early or late manifestations, such as EM rash, cranial nerve palsy, arthritis, or meningitis.

c Post-treatment Lyme symptoms (PTLS) refer to patients with a prior history of clinician diagnosed and treated Lyme disease but who report persistent symptoms that started after Lyme disease and continue despite antibiotic treatment.

#### 2.2.1 Erythema Migrans cases, no prior LD (EM)

Patients with a first episode of LD.

#### *2.2.2* Erythema Migrans cases, with prior LD (EM ​+ ​prior LD)

All patients in this group had a new recent case of EM as well as a prior episode of clinician diagnosed and treated LD. This group is considered to have been reinfected with *Bb*.

#### 2.2.3 PTLS (Post-Treatment Lyme Symptoms) cases

These individuals had been diagnosed with Lyme disease more than 6 months prior to our serum study and reported symptoms that persisted despite prior antibiotic therapy.

#### 2.2.4 Controls

These were individuals without a history of Lyme disease and without a known major medical illness whose serum was negative for *Bb* antibodies on six different Lyme disease assays: Lyme C6 ELISA, Lyme IgM & IgG Western blots, Euroimmune *Bb* ELISA and Euroimmune *Bb* IgM & IgG Immunoblot.

### 2.3 Direct enzyme-linked immunosorbent assay (ELISA)

Ninety-six-well microtiter plates (Greiner Bio-One, Monroe, NC) were coated with 50 ​μL of antigen as follows: 10 ​μg/mL of purified tubulin (MP Biomedicals, Santa Ana, CA), 10 ​μg/mL of dopamine D1 receptor (D1R, PerkinElmer, Waltham, MA), 10 ​μg/mL dopamine D2L receptor (D2R, PerkinElmer), and 20 ​μg/mL of lysoganglioside G_M1_ (Sigma Aldrich, Darmstadt, Germany). Tubulin-, D1R-, and D2R-coated plates were washed 3 times with phosphate buffered saline (PBS, pH 7.2) containing 0.1% Tween (ThermoFisher Scientific, Waltham, MA). Lysoganglioside G_M1_-coated plates were washed 3 times in PBS without Tween in all steps. Plates were blocked with 1% bovine serum albumin (BSA, ThermoFisher Scientific) in PBS for 60 ​min at 37 ​°C. Serum was diluted in 1% bovine serum albumin (BSA, in 1xPBS) then incubated overnight at 4 ​°C. A standard ELISA to detect IgG specific binding to neuronal antigens was performed as previously described (Ben-Pazi et al., 2013; Cox et al., 2013). Samples were assayed in duplicate and averaged. Duplicates not matching with ≥20% variance were repeated. Titers represent the serum dilution at optical density of 0.1 ​at 405 ​nm after 2 ​h. Known positive and negative control samples were included on each plate to standardize and monitor assay performance (Ben-Pazi et al., 2013; Cox et al., 2013). Each new lot of all reagents and antibodies were validated using serum samples with known titers.

### 2.4 Cell culture

SK-N-SH human neuroblastoma cells (Biedler et al., 1973) obtained from American Type Culture Collection (ATCC HTB-11, Manassas, VA) were grown in complete F12-Dulbecco’s Modified Eagle Medium (ThermoFisher Scientific) as previously described (Kirvan et al., 2003). Complete media contained 10% fetal bovine serum (ThermoFisher Scientific) and 1% penicillin-streptomycin antibiotic (ThermoFisher Scientific).

### 2.5 CAMKII activity assay

Assay for CaMKII activity was performed as previously described (Kirvan et al., 2003). Briefly, SK-N-SH cells were plated in 6-well plates at a density of 2.5 million cells/well and incubated overnight in complete F12-Dulbecco’s Modified Eagle Medium, at 37 ​°C with 5% CO2. The next day, cells were serum starved for 30 ​min in serum-free F12 media with 2 ​mM CaCl2, 2 mMKCl, and 0.4 ​mM MgCl2, then stimulated for 30 ​min with patient sera diluted 1:100 in the same media or with media alone (basal control). Cells were harvested and CaMKII activity measured using the CaMKII assay system (Promega, Madison, WI) per manufacturer’s instructions and as previously described (Kirvan et al., 2003). The protein concentration of each sample was used to standardize the CaMKII enzyme activity, and the percentage of specific activity of baseline (basal control) was calculated for each sample where the basal level was set at 100%. All samples were assayed in triplicate. Sera with known high and low CaMKII activity and a basal control were used to standardize the assay. Absorption of sera with anti-IgG beads was shown to significantly reduce CaMKII activation by sera.

### 2.6 Statistical analyses

Statistical significance was defined as a p-value ≤ 0.05 as determined by Mann-Whitney (non-parametric) test for comparison of the median between independent groups. A non-parametric testing approach was used because normality of the distribution of means could not be assumed under the Central Limit Theorem given the non-normal distributions of the measures and the small subgroup sample sizes in all groups except the PTLS group.

## 3 Results

### 3.1 Anti-neuronal autoantibodies measured by direct ELISA: anti-lysoganglioside antibodies are significantly elevated in EM ​+ ​Prior LD and also in PTLS

**Fig. 1:**
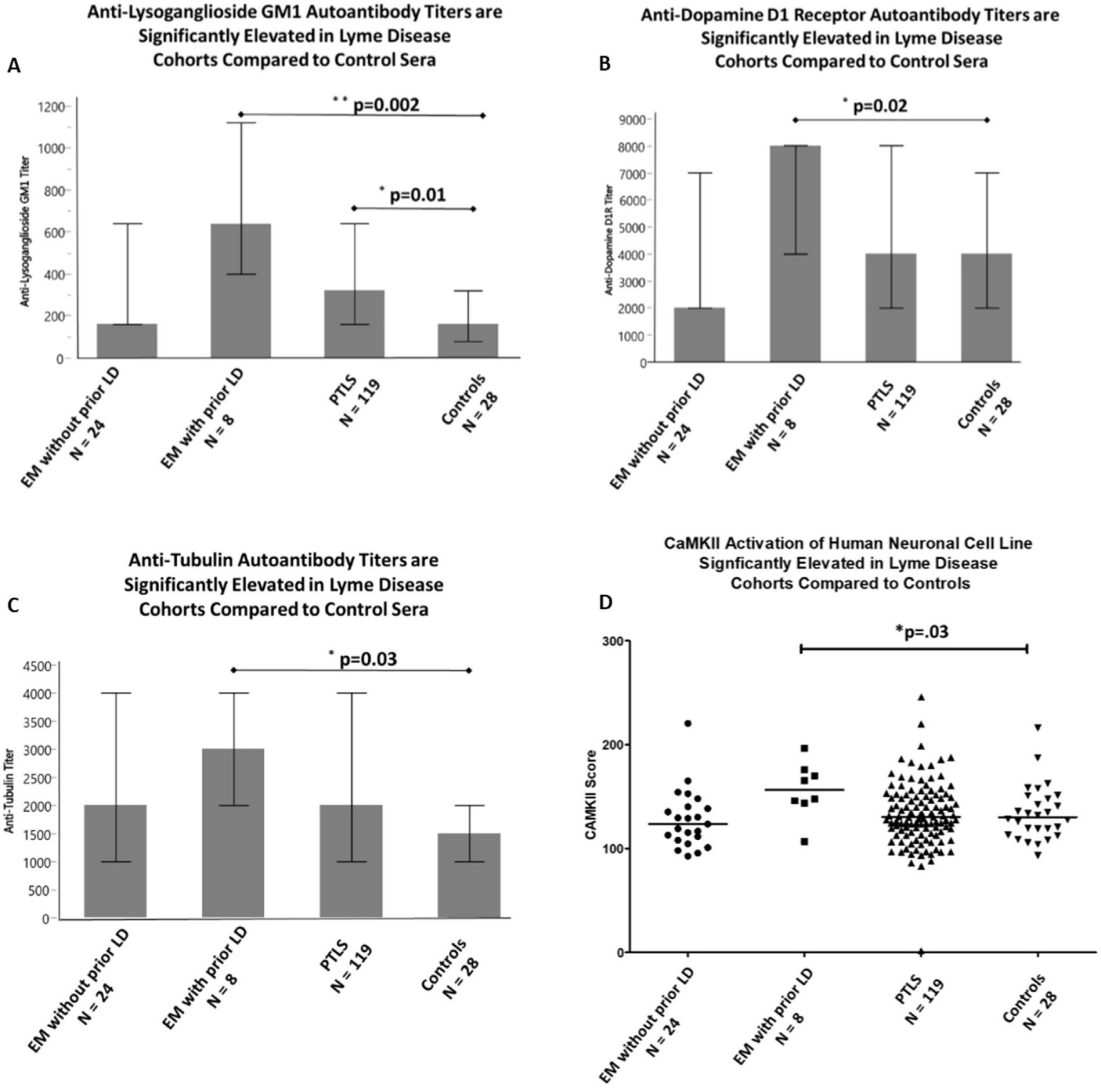
(A) Erythema migrans with prior Lyme Disease and PTLS groups have significantly elevated anti-lysoganglioside GM1autoantibodies compared to the community controls. (B) Compared to controls, Erythema migrans with prior Lyme Disease also have significantly elevated autoantibodies against D1R, (C) and tubulin, (D) and elevated CaM Kinase II activation. [The bar height for figures A–C reflects the median auto-antibody level. Each error bar in the graphs correspond to the 25th and the 75th percentiles.].

Compared to controls, there were no significant differences for any case cohort with respect to anti-D2R median autoantibody titers (Table 2). However, for other autoantibody measures, the EM ​+ ​Prior LD cases had significantly higher titers than controls, including anti-lysoganglioside GM1(p ​= ​0.002) (Fig. 1A), anti-D1R (p ​= ​0.02) (Fig. 1B), and anti-tubulin (p ​= ​0.03) (Fig. 1C). The increase in median titers as shown in Table 2 for the LD case groups ranged from 4-fold for anti-lysoganglioside GM1 (640 vs 160) to 2-fold for anti-D1R (8000 vs 4000) and anti-tubulin (3000 vs 1500). The PTLS group had significantly higher titers of anti-lysoganglioside GM1 antibodies compared to controls (320 versus 160 median titers, p ​= ​0.01). Overall, the most significant and highest titers compared to controls were observed in the EM ​+ ​Prior LD and in the PTLS groups.

**Table 2:** Median anti-neuronal autoantibody titers and serum activated CaM Kinase II activity in human neuronal cells in Lyme disease case groups compared to controls.

Group	Anti-D1R	Anti-Lysoganglioside	Anti-Tubulin	Anti-D2R	CAM Kinase II
EM, no prior LD (n ​= ​24)	2000+	160	2000	8000	124^b^
EM, prior Lyme (n ​= ​8)	8000 p ​= ​0.02^a^	640 p ​= ​0.002	3000 p ​= ​0.03	8000	157 p ​= ​0.03
PTLS (n ​= ​119)	4000	320 p ​= ​0.012	2000	8000	131
Control (n ​= ​28)	4000	160	1500	8000	130

a Mann Whitney (each group vs. control).

b Titer calculated at an endpoint 0.1 optical density and the CaMKII endpoint at 130 percent above the basal rate in human neuronal cell line SKNSH.

Although the sample size is small, the distinction of the EM ​+ ​Prior LD cases is apparent in Fig. 1A–C, which show significantly increased median values on all measures; differences are not due to a few outliers.

### 3.2 LD serum functionally activates CaMKII in human neuronal cell line

The CaMKII assay measures the activation of the enzyme in the human neuronal cell line after treatment with each patient and control serum. The findings in Fig. 1D demonstrate the significantly elevated activation of the CaMKII enzyme in the human neuronal cell line by serum from EM ​+ ​Prior LD with a median of 157 above basal activity (P ​= ​0.03).

It is possible that there was variation in the level of autoantibodies based on the *Bb* serostatus of the individuals within the LD case groups. We examined the distribution of antineuronal autoantibodies and serum activation of CaMKII activity in cases testing negative vs positive for Bb antibodies using two classification methods - the single tier C6 ELISA (C6 ELISA) and the more specific two-tier algorithm (C6 ELISA and EUROLINE –RN-AT IgG) as described previously (Strobino et al., 2018). *Bb* serostatus at the time of sample collection was not associated with significant differences in autoantibodies or CaM kinase activation, regardless of whether serostatus was categorized based on the C6 ELISA alone or the two-tier algorithm. For example, among those with a *negative* C6 test, median titers were 5600, 489, 2550, 7986, 136 for anti-D1R, anti-lysoganglioside GM1, anti-tubulin, anti-D2R, and CAM Kinase II, respectively. In comparison, among those with a *positive* C6 test, mean titers were 5965, 480, 3180, 8900, and 131, respectively.

## 4 Discussion

Autoantibodies may develop after infections that target human tissue causing neurologic and neuropsychiatric disease (Ben-Pazi et al., 2013; Tatsumoto et al., 2006). Given that anti-neuronal autoantibodies may play a role in streptococcal neurological sequelae, we tested the hypothesis that anti-neuronal autoantibodies might also be elevated in LD.

We discovered that patients with a history of prior LD who were subsequently reinfected with *Bb* exhibited a distinct elevation of their anti-neuronal antibody response which differentiated them from the cohort of newly infected patients without prior LD, as well as from the larger group of patients with a history of persistent symptoms despite prior treatment. The serum of these patients also led to an elevated CaMKII response, suggesting that the anti-neuronal antibodies have functional significance. Whether antineuronal antibodies and CaMKII response are associated with clinical symptoms in our sample remains unclear, as our study did not assess clinical severity. However, in other diseases such as Sydenham chorea, a correlation between CaMKII activation and symptoms has been demonstrated (Kirvan et al., 2003, 2006).

It is reasonable to hypothesize that neurologic or neuropsychiatric symptoms may emerge as a result of elevated autoantibodies against lysoganglioside GM1, a well-known target of neuronal antibodies in disease, and against the dopamine 1 receptor, both leading to activation of CaMKII in neuronal cells. Evidence supporting functional significance comes from studies showing that depletion of IgG removes the CaMKII activation of neuronal cells by sera (Brimberg et al., 2012). A heightened immunological response triggered by repeated infection with *Bb* may increase the risk for neurologic and neuropsychiatric symptoms among patients with previously treated LD.

The anti-lysoganglioside GM1 antibody was elevated also in the post-treatment LD group. Ganglioside is a molecule that is very common in the brain, helping to modulate cell signal transduction (Kolter, 2012). GM1 ganglioside expression is necessary for axonal growth and healthy cognitive function and is diminished in other central neurologic diseases (Rubovitch et al., 2017).These gangliosides also serve as receptors for pathogenic bacteria and viruses, such as Helicobacter pylori, Vibrio cholerae (toxin), Clostridium tetani (toxin), and Plasmodium falciparum. Animal studies have demonstrated that immunization with *Bb* produced IgM antibodies that cross-react with gangliosides (asialo-GM1 and GM1) (Garcia-Monco et al., 1995). Human studies have shown elevated levels of anti-ganglioside antibodies in Lyme neuroborreliosis, autoimmune-mediated peripheral neuropathies, connective tissue diseases, and in selected autoimmune encephalitides (García-Moncó et al., 1993; Tatsumoto et al., 2006; Weiner et al., 2000).

Our study adds to recent evidence indicating that anti-neuronal antibodies may play a role in human LD and suggests that repeated infection may increase the risk of developing anti-neuronal antibodies. A particular strength of this study is that our controls came from the same Lyme endemic areas as the patients and were screened to be free of *Bb* antibodies using 6 different assays to ensure no prior *Bb* exposure. A study limitation relating to the patient cohorts is that we do not know whether antineuronal autoantibodies were present prior to the *Bb* infection or whether their emergence coincided with post-treatment symptoms. Second, our sample size of those with recent EM plus prior infection was small. Third, because we did not inquire about functional status, we employ the term “Post-treatment Lyme Symptoms” rather than PTLDS. Because PTLDS requires functional impairment, it is possible that the antineuronal antibody levels and the CaM Kinase II activation profile might be more pronounced in a pure PTLDS cohort. Fourth, our screening questionnaire at the time of serum collection did not include measures of current symptom severity, thereby precluding an assessment of whether the presence of these autoantibodies or the Cam Kinase II activation relate to current clinical symptoms. Fifth, it is possible that the cohort of patients with current EM and prior LD also by chance had a higher frequency of prior infection with GABHS, thereby accounting for their elevated antineuronal autoantibody titers and the CaM Kinase II activation. We however do not think this is likely as nearly all adults have had prior infection with GABHS and thus the distribution of prior infection should be similar across cohorts; nevertheless, this could be readily tested in a future study. Finally, the PTLS group may have contained some individuals who were treated for “possible” rather than “confirmed” LD. Given that group heterogeneity limits the power of a study to find biomarkers of a disease, it is remarkable that we nevertheless did find significantly elevated levels of the anti-lysoganglioside autoantibody in the PTLS group, as we had in the group with a new EM and history of previously treated LD. This raises the possibility that elevated anti-lysoganglioside antibody levels may play a role in symptom persistence. Our hypothesis is that after a second *Bb* infection, the elevation of the autoantibodies would continue to increase and persist in the patients who would be at risk of developing or who do develop long term symptoms. Our results further support previous findings in Lyme disease of elevated anti-ganglioside antibodies and provides a basis for future studies of autoantibodies related to the neurologic, neuropsychiatric, and cognitive deficits associated with early and later stages of Lyme disease.

Future studies should evaluate whether a correlation exists between symptom emergence, symptom severity, and anti-neuronal antibody levels. If a significant correlation is discovered, then immunomodulatory therapy should be investigated as a potential treatment option for PTLS; favorable results have been reported in a case series of *Bb*-triggered small fiber neuropathy (Katz and Berkley, 2009) and in case reports of *Bb*-triggered Guillain-Barre (Patel et al., 2017) and immune-mediated neuropathy (Rupprecht et al., 2008). Future prospective studies of acute LD should include those with and without a prior history of LD to determine whether there is an immune-priming response that may increase the likelihood of developing *Bb*-triggered autoimmune disease as has been suggested by our data.

## 5 Conclusion

The finding of elevated anti-neuronal autoantibodies in our small sample of those with a prior history of Lyme Disease, if replicated in a larger sample, suggests an immune priming effect of repeated infection; the CaMKII activation suggests that antineuronal antibodies have functional significance. The elevation of anti-lysoganglioside antibodies among those with PTLS is of particular interest given the established role of anti-ganglioside antibodies in peripheral and central neurologic diseases.

## Declaration of competing interest

The authors declare the following financial interests/personal relationships which may be considered as potential competing interests: None for B. Fallon, B. Strobino, S.Reim or J. Stoner. Madeleine Cunningham is chief scientific officer and co-founder with financial interest in Moleculera Labs, a commercial laboratory for diagnostic testing of autoantibodies against the heart and brain.
